# Effects of changes in diet energy density and milking frequency and a single injection of cabergoline at dry-off on feeding behavior and rumination time in dairy cows

**DOI:** 10.3168/jdsc.2021-0162

**Published:** 2022-03-03

**Authors:** G.A. Franchi, M.L.V. Larsen, M.S. Herskin, L. Foldager, M. Larsen, M.B. Jensen

**Affiliations:** 1 Department of Animal Science, Aarhus University, Blichers Allé 20, 8830 Tjele, Denmark; 2 Bioinformatics Research Centre, Aarhus University, C.F. Møllers Allé 8, 8000 Aarhus, Denmark

## Abstract

Dry-off is a typical management practice, but research on the effects of dry-off on feeding behavior in high-yielding cows is limited. The present study investigated the effects of 2 diet energy densities: lactation diet (normal energy density, NORM) versus a lactation diet diluted with 30% straw (reduced energy density, REDU), both offered ad libitum, and 2 daily milking frequencies (2× vs. 1×) during the 7 d before dry-off day (d 0), and the effects of an injection of either a dopamine agonist [cabergoline (CAB); Velactis, Ceva Santé Animale; labeled for use only with abrupt dry-off; i.e., no reduction in feeding level or milking frequency before the last milking] or saline (SAL) following the last milking on d 0 (2 × 2 × 2 factorial arrangement), on automatically monitored feeding behavior and rumination time in 119 clinically healthy, loose-housed, pregnant, lactating Holstein cows during the week before and after d 0. From d 0, all cows were fed the same dry-cow diet ad libitum. Data were analyzed in R using mixed-effects models. Over the days before d 0, REDU cows spent 30% more time feeding at a 50% lower feeding rate, visited both assigned and unassigned feed bins more frequently, and spent more time ruminating than NORM cows. No clear behavioral effects of reduced milking frequency were found. Within 24 h following injection, CAB cows spent approximately 40% less time feeding at a lower feeding rate, visited their feed bin 28% less often, and spent 40% less time ruminating than SAL cows, irrespective of treatment before dry-off. The current study demonstrates that reducing diet energy density for 1 wk before dry-off led to clear behavioral changes in high-yielding cows. Administering CAB after the last milking induced decreased feeding behavior lasting approximately 24 h, indicating collateral effects other than reduced prolactin secretion.

## Highlights

• Diluting the lactation diet with straw induced feeding behavior changes.
• Behavioral effects of gradual milking cessation were unclear.
• Cabergoline induced a reduction in feeding behavior lasting approximately 24 h.

## Graphical Abstract

**Figure:**
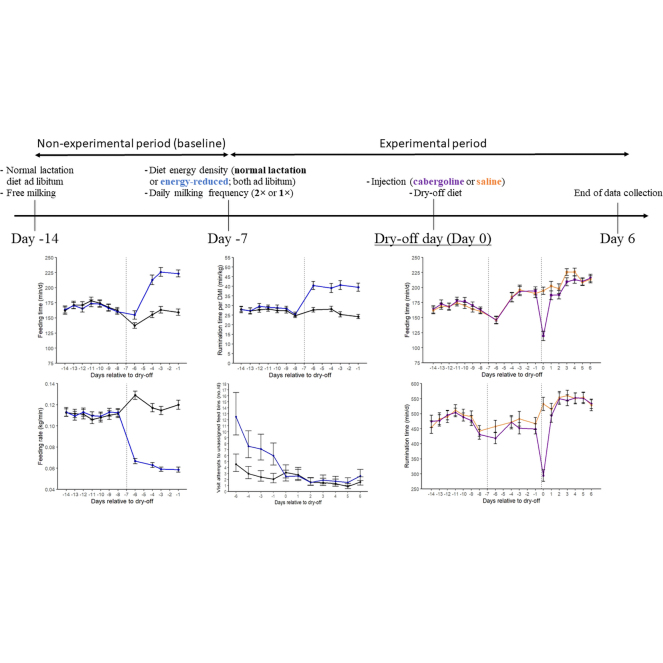
**Summary:** Typically, dairy cows have their milk production artificially ceased generally 60 d before the date of expected calving in a process termed “dry-off.” Dry-off can be abrupt; that is, diet and milking frequency remain unchanged until the day of last milking, or gradual, aiming to reduce milk yield before the onset of the dry period. Abrupt dry-off may be facilitated by administration of prolactin-release inhibitors such as cabergoline. We examined the effects of dietary and milking frequency changes during 1 wk before the dry-off day, as well as injection of cabergoline following the last milking, on automatically monitored feeding behavior and rumination time in high-yielding (.15 kg/d at dry-off) cows. Reducing diet energy content by diluting the lactation diet with straw induced consistent feeding behavior changes before dry-off. The effects of gradual milking cessation were unclear, while cabergoline reduced feeding behavior for approximately 24 h.

In most commercial dairy farms, milk production is artificially stopped approximately 2 mo before the expected calving date in a process called “drying-off” (Capuco et al., 1997). The dry period allows cows a resting period and stimulates renewal of udder cells, which will potentially lead to higher milk production in the next lactation (Andersen et al., 2005). Some farmers dry off cows abruptly (Zobel et al., 2015). Alternatively, drying-off can be gradual, comprising dietary (quantitative or qualitative) and daily milking frequency changes before the last milking (i.e., the dry-off day). Gradual dry-off aims to reduce milk production and minimize the risks of milk leakage and IMI due to milk accumulating in the udder in the early dry period (Zobel et al., 2015; Vilar and Rajala-Schultz, 2020). Additionally, cessation of milking during an abrupt dry-off may be facilitated by administration of dopamine agonists, such as cabergoline (Boutinaud et al., 2016; Bertulat et al., 2017). Irrespective of method, drying-off can induce behavioral changes in high-yielding cows (≥15 kg/d on the day of last milking; Vilar and Rajala-Schultz, 2020). For instance, restricting energy supply to the udder by feeding forage-rich diets may result in changes in feeding behavior such as increased feeding time and rumination and decreased feeding rate (Valizaheh et al., 2008; Yang and Beauchemin, 2009; Dancy et al., 2019). Furthermore, as prolactin secretion is mostly controlled by the hypothalamus and dopaminergic neurons, which are also involved in the modulation of ruminal motility and central reward processing, among other biological processes (Fioramonti and Bueno, 1988; Martins et al., 2017), the administration of cabergoline may collaterally affect feeding behavior and rumination of dairy cows.

To our knowledge, only 2 studies (Valizaheh et al., 2008; Dancy et al., 2019) have investigated the effects of reducing energy supply at dry-off on the feeding behavior of dairy cows. However, neither investigated the behavioral effects of dietary change combined with milking frequency changes. Moreover, limited data are available on the effects of cabergoline on DMI in drying-off cows (Larsen et al., 2021), and investigations of cabergoline's effects on feeding behavior are lacking. Accordingly, we aimed to investigate the single and combined effects of dietary and milking frequency changes during the week before dry-off, as well as the effect of cabergoline following the last milking on the dry-off day, on automatically monitored feeding behavior and rumination time of high-yielding cows. We hypothesized that reducing energy supply before dry-off by diluting the lactation diet with straw would increase time spent feeding and ruminating and decrease the feeding rate because of changes in physical characteristics of the diet. Given that twice-daily-milked cows fed a low-energy diet were in negative energy balance (**NEB**) before dry-off (Larsen et al., 2021), we hypothesized that these changes would be more pronounced in these cows. Moreover, we hypothesized that the administration of cabergoline would collaterally reduce feeding behavior in cows.

All procedures involving animals were approved by the Danish Animal Experiments Inspectorate (Permit No. 2017-15-0201-01230) in accordance with the Danish Ministry of Environment and Food Act No. 474 (May 15, 2014). The experimental work was conducted according to Good Clinical Practice Guideline VICH GL19 (VICH, 2001), and the unregistered use of cabergoline (Velactis, Ceva Santé Animale) was approved by the Danish Medical Agency (Permit No. 2017064040). In countries where Velactis is registered, Velactis is labeled to be used with abrupt dry-off; that is, no reduction in feeding level or milking frequency before the last milking. In other dry-off regimens, use of Velactis is off-label.

The experiment was described in detail by Larsen et al. (2021). In brief, this study included 119 (72 primi- and 47 multiparous) loose-housed, lactating and pregnant Holstein cows in a randomized block design with repeated measurements. Cows from the resident herd were continuously enrolled into blocks of 8 within parity group, 14 d before the dry-off day (d 0). Enrollment occurred every 2 wk in batches of 1 to 6 cows, depending on the availability of cows, resulting in 36 successive batches (in 3 batches, only 1 experimental cow was enrolled and was therefore housed with a nonexperimental companion cow). Hence, 2 to 6 cows were housed in an experimental home pen in the same barn as the resident pens from d −7 to 7 relative to d 0. The experimental pen consisted of an 8.6- × 4-m feeding area equipped with 6 automated feed bins (Insentec B.V.; validated by Chapinal et al., 2007), and an 8- × 2.6-m alley with 10 cubicles. Experimental cows were herded via a corridor to be milked in an automatic milking system (**AMS**; DeLaval AB).

Between d −14 and −8, cows were fed a partially mixed normal lactation diet (**NORM**; Table 1) in automated feed bins and were freely milked in the AMS. During milking, cows were allowed a maximum of 3 kg/d of a commercial pelleted concentrate (Table 1). From d −7 to −1, treatments followed a 2 × 2 factorial arrangement with diet energy density [NORM diet or energy-reduced diet (**REDU**; Table 1), both fed ad libitum] and daily milking frequency [2× (0530–0700 h and 1530–1630 h) or 1× (0530–0700 h)]. Feed delivery occurred 4 times daily (at approximately 0630, 1030, 1430, and 2000 h) following routine barn management. During the week before dry-off, the amounts of feed in each bin after feed delivery (i.e., automatically recorded wet weight following feed bin opening upon the end of the feed delivery) for NORM and REDU cows were (mean ± SD) 32 ± 9 and 21 ± 6 kg, respectively. After dry-off, the amount of feed in each bin after feed delivery was 23 ± 6 kg. Feed bins were emptied on Mondays, Wednesdays, and Fridays at approximately 1000 h. The amounts of feed emptied out (i.e., automatically recorded wet weight following feed bin closing for cleaning) from feed bins assigned to NORM and REDU cows were 9 ± 4 and 8 ± 4 kg, respectively. After dry-off, the amount of feed emptied out from feed bins was 9 ± 5 kg. During milking, cows were offered either a maximum of 3 kg/d (NORM diet) or 1 kg/d (REDU diet) of concentrate, with an equal allowance at each milking for the 2× milking frequency. The amounts of concentrate offered and refused at each milking were recorded in the AMS. Feed mixes were sampled weekly; samples were pooled within 5-mo periods and analyzed (n = 4; Table 1) for chemical composition, as described by Larsen et al. (2021), and for particle size distribution, as described by Kononoff et al. (2003).

**Table 1:** Composition of normal lactation diet (NORM), energy-reduced lactation diet (REDU), dry-cow diet, and concentrate (g/kg of DM unless otherwise noted; retrieved from Larsen et al., 2021)

Item	Lactation cow feed			Dry-cow diet
	NORM	REDU	Concentrate^1^	
Ingredient				
Maize silage	325.4	205.3	—	528.5
Grass-clover silage	282.1	178.0	—	132.2
Straw, barley	—	302.7	—	246.7
Barley grain	104.1	65.7	141.5	—
Wheat grain	—	—	141.5	—
Sugar beet pulp, dried	104.1	65.7	172.5	—
Rapeseed cakes	86.8	54.8	—	35.3
Rapeseed meal	—	—	169.3	—
Soybean meal, 54% CP	86.8	54.8	90.8	35.3
Citrus pulp, dried	—	—	72.5	—
Sunflower meal	—	—	71.0	—
Grass pellets	—	—	52.4	—
Wheat bran	—	—	49.6	—
Molasses, sugar beet	—	—	18.6	—
Vegetable fat, saturated	—	—	9.0	—
Mineral and vitamin premixes	5.5^2^, ^3^	13.0^2^, ^3^	2.3^4^	9.7^5^
NaHCO_3_	2.4	7.8	—	—
NaCl	1.7	7.4	8.0	3.5
CaCO_3_	1.1	7.0	—	4.4
Ca(H_2_PO_4_)_2_	—	31.5	—	4.4
MgO	—	6.3	—	—
MgSO_4_	—	—	1.0	—
Nutrient (mean ± SD)				
DM (g/kg)	396 ± 23	455 ± 27	901 ± 2	407 ± 26
Ash	70 ± 3	106 ± 4	66 ± 3	64 ± 3
CP	168 ± 3	124 ± 4	214 ± 1	115 ± 9
Crude fat	37 ± 2	27 ± 1	35 ± 2	28 ± 0.5
NDF	312 ± 8	429 ± 16	235 ± 4	443 ± 25
Starch	172 ± 5	108 ± 9	199 ± 10	188 ± 10
NEL_20_ (MJ/kg of DM)^6^	6.59	4.96	6.76	5.40
Particle size distribution (g/kg; mean ± SD)				
>19.0 mm	140 ± 31	330 ± 21	—	278 ± 24
19.0–8.0 mm	409 ± 17	273 ± 10	—	439 ± 40
8.0–1.18 mm	373 ± 30	310 ± 14	—	225 ± 10
<1.18 mm	78 ± 17	87 ± 2	—	58 ± 13

1 Commercial pelletized concentrate (SL395044, DLG).

2 Mineral and vitamin premix lactation (Type 3, ViloFoss).

3 E-vitamin premix (Suplex E-50000, ViloFoss).

4 Micro-mineral and vitamin premix (ViloFoss).

5 Mineral and vitamin premix dry (Komix Top Gold d-alfa Org Se, ViloFoss).

6 Standard feed value for NE_L_ at 20 kg of DMI calculated according to NorFor (Volden, 2011).

After the last milking on d 0, the study followed a 2 × 2 × 2 factorial arrangement with the inclusion of the third factor: a single i.m. injection of cabergoline (**CAB**) or saline (**SAL**). After dry-off, all experimental cows were fed a dry-cow diet ad libitum (Table 1). Cows were the experimental unit because treatments were applied at this level. A planned sample size of 15 cows/treatment was based on the availability of cows in the resident herd and supported by power calculations to detect significant differences (at 5% significance level) between NORM and REDU cows for feeding time (mean ± SD: 211.1 ± 23.8 vs. 247.7 ± 23.8 min/d), feeding rate (0.12 ± 0.015 vs. 0.07 ± 0.015 kg of DM/min), and rumination time per DMI (20.6 ± 2.8 vs. 29.3 ± 2.8 min/kg of DM) with a power of at least 90% using the R package *pwr* v.1.3–0 (Champely, 2020). These mean differences and SDs were calculated from Table 4 in Dancy et al. (2019) at baseline (closest to our NORM diet) and for a lower nutrient density diet treatment group (closest to our REDU diet), using the largest SD. Two cows became lame (1 cow before d −14 and 1 cow on d −7) and 2 cows showed clinical symptoms of mastitis (1 cow on d −7 and 1 cow on d −5); therefore, they were excluded from the entire study. Hence, a total of 115 cows were included in the data set (Table 2). On d −14, included cows weighed 778 ± 77 kg, were 225 ± 6 d in pregnancy, and yielded 26 ± 6 kg/d.

**Table 2:** Breakdown of the number of cows across treatments included in the study as well as the final number of cows across treatments included in the feeding behavior and rumination time data sets

Treatment			Data sets			Microphone- or accelerometer-based sensor^3^ (n/n)
Energy density^1^	Milking frequency	Injection^2^	Total cows included (n)	Feeding behavior	Rumination time	
NORM	2×	SAL	14	14	13	7/6
		CAB	14	14	10	7/3
	1×	SAL	15	15	11	7/4
		CAB	14	14	10	6/4
REDU	2×	SAL	14	14	10	6/4
		CAB	15	14^4^	10	6/4
	1×	SAL	15	15	7	4/3
		CAB	14	13^4^	13	8/5
Total number			115	113^4^	84^5^	51/33

1 NORM = normal lactation diet; REDU = energy-reduced lactation diet.

2 SAL = saline; CAB = cabergoline.

3 Microphone- or accelerometer-based Heatime Ruminact (SCR Engineers Ltd.) sensor attached to the left side of the neck collar of each experimental cow.

4 One cow in each treatment had no access to the feed bin for 18 h between d −1 and 0.

5 Removal of cows across treatments were due to equipment failure to register twelve 2-h intervals daily over experimental days.

Feeding behavior of each cow was automatically recorded by the automated feed bins. From d −14 to −8, in the resident herd, 2 to 3 cows shared 1 feed bin. From d −7 to 7, cows were randomly assigned a specific feed bin (1 bin/cow). A visit to a feed bin was defined from the moment the cow put her head inside the feed bin while placing her collar past the feed bin gate and opening the gate until she removed her head from inside the feed bin area. At each visit to the assigned feed bin, visit duration (i.e., an estimate of feeding time) and amount of feed consumed (start weight minus end weight; as-fed intake) were recorded. The DMI of each feed was calculated by multiplying the as-fed intake from each feed bin visit by the respective DM percentage (Larsen et al., 2021). Subsequently, feeding rate (kg of DM/min) was calculated as DMI (kg/d) divided by feeding time (min/d). In addition, visit attempts to unassigned feed bins (i.e., cow put her head inside any unassigned feed bin and the gate did not open) were recorded from d −7 to 6. Data were aggregated to day level with a day running from 1000 h on the actual day to 0959 h on the day after (because treatments were initiated between 0800 and 1000 h on d −7 and 0). Before data aggregation, outliers were identified and handled as described in Larsen et al. (2021). Briefly, visits lasting 0 s, visits with end time occurring later than the start time of the subsequent visit, and visits lasting longer than 30 min were deleted (166 visits). Visits with feeding rate deviating more than 7 × SD of the cow's weekly average, below the 0.025% quantile, or above 99.975% quantile were replaced with the weekly median for each cow (326 visits). The feeding behavior data set included 2,010 daily observations of 113 cows (Table 2).

Rumination time was recorded from d −14 to 6 using the sensor Heatime Ruminact (SCR Engineers Ltd.) attached to the left side of the neck collar of each experimental cow as validated by Schirmann et al. (2009), being 61% microphone-based and 39% accelerometer-based sensors (Table 2). Rumination data were summed and saved at 2-h intervals. Hence, absolute rumination time per cow (min/d) was the sum of twelve 2-h intervals. If fewer than twelve 2-h intervals were registered, the daily observation was considered missing. Rumination time per DMI (min/kg of DM) of each cow was calculated as rumination time divided by daily DMI to account for differences in feed intake between the treatments. In both data sets, observations recorded on d −7 (Wednesday), −5 (Friday), and −2 (Monday) were removed because 60 cows were participating in behavioral tests outside the experimental home pen (Franchi et al., 2019, 2020). Rumination time data comprised 1,455 daily observations of 84 cows (Table 2).

Data were analyzed in R v.4.0.3 (R Core Team, 2020). The baseline average (d −14 to −8) was tested for differences among treatment combinations using a general linear model with parity group (primiparous, multiparous) and treatment combination (1–8) as fixed effects. Unless otherwise stated, whenever mixed-effects modeling was applied (*glmmTMB* v.1.0.2.1; Brooks et al., 2017), the fixed effects were parity group, days relative to the dry-off day (−6, −4, −3, −1, 0, 1, 2, 3, 4, 5, 6), diet energy density (NORM, REDU), daily milking frequency (2×, 1×), type of injection (SAL, CAB) and all possible 2-, 3-, and 4-way interactions between diet energy density, daily milking frequency, type of injection, and day relative to dry-off. Number of cows in the pen (2–6) and baseline average were included as covariates. Batch and cow were included as random effects. Additionally, a continuous-time autoregressive Ornstein-Uhlenbeck correlation structure was included to account for repeated measures of each cow over days. Post hoc analyses were performed with Tukey-adjusted least squares means (*emmeans* v.1.5.0; Lenth, 2020). Model assumptions of normality and homoscedasticity were confirmed through graphical inspection of the residuals. Only results (least squares means ± standard errors) of effects at *P-*value < 0.1 are presented and discussed herein.

Feeding time and feeding rate were analyzed with Gaussian linear mixed-effects models. Frequency of visits and frequency of visit attempts were analyzed with negative bionomial generalized linear mixed-effects models with a log-link function. The fit of the visit attempts model was not initially confirmed. Therefore, the 4-way interaction was removed. Afterward, the fit of the reduced model was reassessed and not rejected. Rumination time data were analyzed with Gaussian linear mixed-effects modeling as described above, including sensor type (microphone, accelerometer) as fixed effect. No effect of sensor type was detected on rumination time data, in either minutes/day (χ^2^_1_ = 0.4, *P* = 0.544) or in minutes per kilogram of DM (χ^2^_1_ = 2.5, *P* = 0.114). Furthermore, absolute rumination time was analyzed at the 2-h level for 48 h from 1000 h on d −1 to 0959 h on d 1. The analysis was performed with a Gaussian linear mixed-effects model including sensor type, type of injection, 2-h interval, and 2-way interaction between type of injection and 2-h interval as fixed effects. Batch and cow were included as random effects. The average baseline over d −6, −4, and −3 was included as covariate. The model contained a continuous-time autoregressive covariance structure of order 1 to account for repeated measures of each cow over 2-h intervals.

For variables recorded in the baseline period, no differences among treatments were observed. Over d −6, −4, −3 and −1, REDU cows spent more time feeding (204 ± 3.9 vs. 154 ± 2.6 min/d; χ^2^_10_ = 107.1; *P* < 0.001), had a 50% lower feeding rate (0.06 ± 0.001 vs. 0.12 ± 0.002 kg of DM/min; χ^2^_10_ = 1004.3; *P* < 0.001), visited their feed bin approximately 30% more often (34 ± 1.0 vs. 24 ± 0.8 no./d; χ^2^_10_ = 30.9; *P* < 0.001), and attempted to visit unassigned feed bins more than twice as often (11 ± 0.8 vs. 4 ± 0.3 no./d; χ^2^_10_ = 62.1, *P* < 0.001) compared with NORM cows. Additionally, REDU cows spent, on average, more time ruminating than NORM cows, both in absolute terms (515 ± 8.2 vs. 490 ± 7.8 min/d; χ^2^_10_ = 33.8; *P* < 0.001) and per DMI (42 ± 1.1 vs. 35 ± 1.1 min/kg of DM; χ^2^_10_ = 86.1; *P* < 0.001).

Cows administered CAB spent less time feeding on d 0 (122 ± 6.0 vs. 197 ± 5.9 min/d) and 1 (188 ± 6.0 vs. 203 ± 5.9 min/d; χ^2^_10_ = 153.8; *P* < 0.001), displayed lower feeding rate on d 0 (0.06 ± 0.002 vs. 0.07 ± 0.002 min/kg of DM; χ^2^_10_ = 35.3; *P* < 0.001), and visited their feed bin approximately 28% less often on d 0 (21 ± 1.2 vs. 27 ± 1.4 no./d; χ^2^_10_ = 51.0; *P* < 0.001) than SAL cows. Additionally, on d 0, CAB cows spent over 40% less time ruminating than SAL cows (308 ± 12.2 vs. 535 ± 12.9 min/d; χ^2^_10_ = 276.8; *P* < 0.001), with an average nadir of 7 min/2-h interval at 3 to 5 h after injection (χ^2^_23_ = 189.2; *P* < 0.001; Figure 1). Effects of milking frequency change were unclear and only detected after dry-off. On d 1, among NORM cows, feeding time was longer in 1× cows than 2× cows (210 ± 7.9 vs. 176 ± 8.0 min/d; χ^2^_10_ = 19.8; *P* = 0.030). On d 1 and 5, 1×-CAB cows (45 ± 2.1 and 48 ± 2.0 min/kg of DM) had the longest rumination time per DMI, followed by 2×-CAB cows (44 ± 2.1 and 41 ± 2.1 min/kg of DM), 2×-SAL cows (42 ± 2.1 and 41 ± 2.1 min/kg of DM), and 1×-SAL cows (36 ± 2.3 and 40 ± 2.3 min/kg of DM; χ^2^_10_ = 29.3; *P* = 0.001).

**Figure 1:**
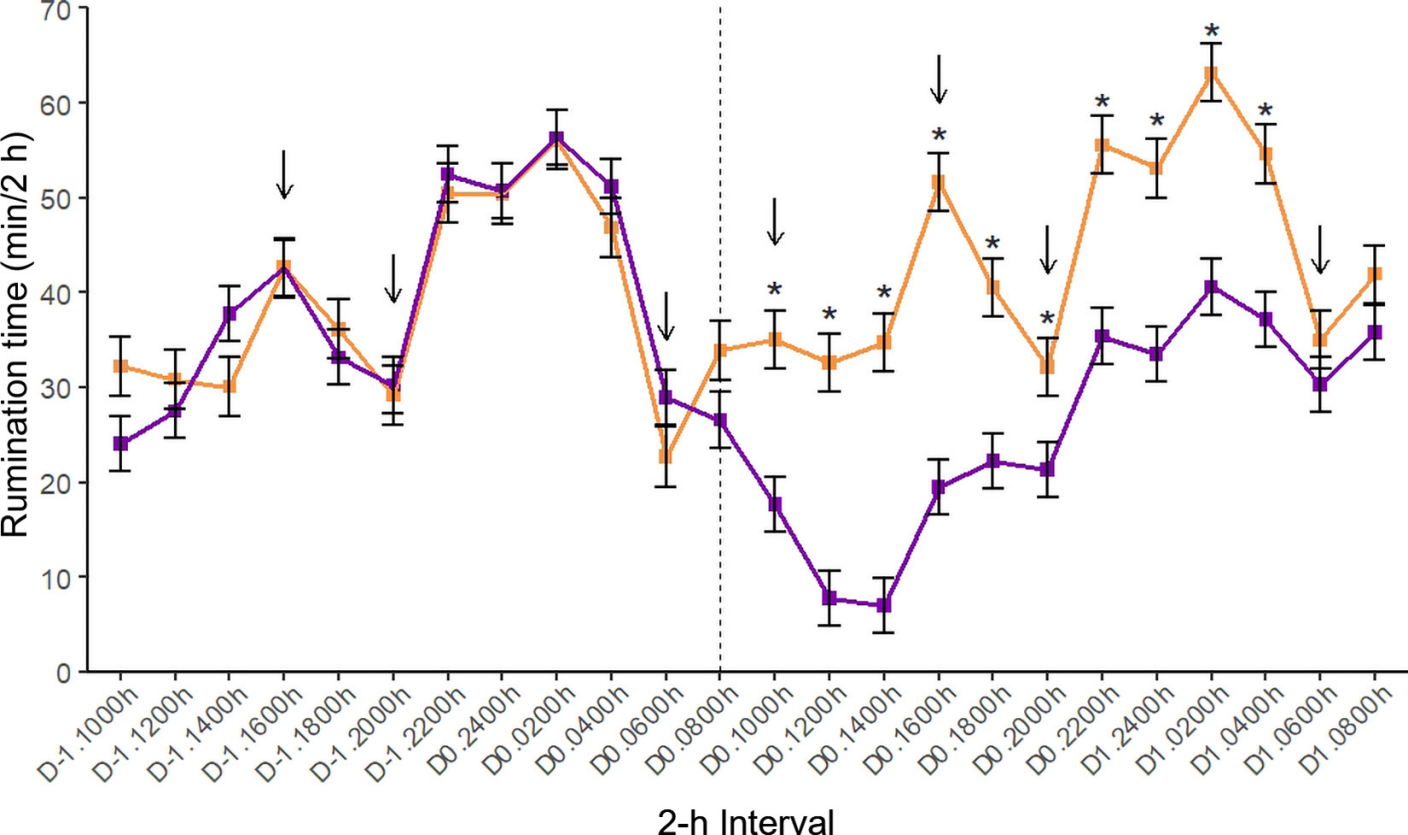
Mean absolute rumination time (min/2-h interval) in cows injected with saline (orange; n = 41) or cabergoline (purple; n = 43) during 48 h from d −1 to 1 (D-1 to D1) relative to dry-off. The vertical dashed line indicates the approximate time when cows were injected after the last milking on dry-off day. The arrows indicate the approximate times when fresh feed was delivered. Asterisks (*) indicate statistical difference between saline and cabergoline cows at *P* < 0.05. Error bars indicate SE.

The current study demonstrated that typical dry-off management practices, especially reducing diet energy concentration through straw dilution of the lactation diet for a week before last milking, can induce changes in feeding behavior. The increased feeding time and decreased feeding rate in cows fed the REDU diet before dry-off was consistent with previous studies examining the effects of low energy diet providing during dry-off (e.g., Valizaheh et al., 2008; Dancy et al., 2019). Further, the longer rumination time per kilogram of DMI in REDU cows reflected higher forage and NDF contents, as well as a greater proportion of long feed particles, of the REDU diet compared with the NORM diet and that ruminal physical fill was likely greater, which lowered voluntary DMI (Yang and Beauchemin, 2009; Jiang et al., 2017). In fact, Larsen et al. (2021) found that NORM cows had an approximately 50% higher DMI and energy consumption than REDU cows. Knowing that REDU cows displayed a consistently increased feeding motivation during the week before dry-off when subjected to 2 feed-thwarting tests (Franchi et al., 2021) and to 2 different feeding motivation tests (Franchi et al., 2019, 2020), we speculate that the increased frequency of visits to both assigned and unassigned feed bins observed in this study reflected the search of REDU cows for additional, or alternative, feed to fulfill their energy requirement. For instance, Moore and DeVries (2020) reported that dairy cows under NEB sort more in favor of smaller, more energy-dense diet components and against longer, less energy-dense particles. Hence, these findings suggest that cows can change their feeding behavior to achieve metabolic homeostasis and maximize nutrient consumption. Additionally, we should not disregard the potential effect of the feed bin cleaning routine on cows' feeding behavior. Indeed, the presence of feed more than 24 h old on days when bins were not emptied may have affected feeding behavior and feed consumption.

At the dry-off day, cows that were abruptly dried off yielded around 25 kg/d of milk, whereas cows gradually dried off by either feeding REDU diet or being milked 1× yielded approximately 30% less, and cows gradually dried off by feeding the REDU diet and being milked 1× yielded approximately 45% less (Larsen et al., 2021). However, no clear effects of reduced milking frequency, singly or in combination with dietary changes, on feeding behavior were detected in the present study. For instance, knowing that 2× cows fed the REDU diet had the most pronounced NEB among the 4 treatment combinations before dry-off (Larsen et al., 2021), we might expect that these cows would display the highest frequency of attempts to visit unassigned feed bins. The absence of clear effects of daily milking frequency changes on feeding behavior is in alignment with the unclear effects of this management practice on feeding motivation measures reported in other parts of the project (Franchi et al., 2019, 2020, 2021). Given that no power calculations were performed to detect significant differences between the 2 milking frequency groups for any of the examined variables, this study may have not been adequately powered to detect milking frequency change effect on feeding behavior.

The administration of CAB to inhibit prolactin secretion collaterally induced a marked reduction in DMI (Larsen et al., 2021) as well as feeding time, feeding rate, frequency of visits to the assigned feed bin, and rumination time lasting approximately 24 h, regardless of treatment before dry-off. These results suggest a temporary systemic response to CAB similar to feeding behavior changes reported in lactating cows challenged by, for instance, intramammary LPS infusion or ruminal acidosis (e.g., Aditya et al., 2017). We propose 2 nonexclusive explanations for these findings. First, the apparent reduction in feeding behavior might have been due to a constrained gastrointestinal motility, which consequently limited feed passage rate and DMI (Fioramonti and Bueno, 1988; Van Miert and Van Duin, 1991). Second, feeding behavior might have declined in response to a reduced perception of feed and general activity (Freeman et al., 2000; Martins et al., 2017). Further studies including additional physiological, behavioral, clinical, and cognitive measures are needed to clarify all potential effects of CAB on drying-off cows.

We encourage future studies examining the effects of these dry-off treatments on cows' feeding behavior to include extra feeding behavior measures, which could not be recorded in the present study. For instance, knowledge about the particle size distribution of feed left in the feed bins would have allowed for investigation of feed sorting, which positively correlates with feeding time and negatively correlates with feeding rate (Greter and DeVries, 2011; Miller-Cushon and DeVries, 2017; Dancy et al., 2019). Additionally, changes in feeding rate and rumination time could have been followed by changes in meal patterns (Jiang et al., 2017; Dancy et al., 2019). For example, as REDU cows spent increased time ruminating (per DMI), the duration of nonfeeding time within meal and total daily meal time would likely be longer than that in NORM cows.

The current study demonstrated that diluting the lactation diet with straw in the week before dry-off, leading to changes in physical characteristics of the diet and reduction of diet energy concentration, clearly induced changes in feeding behavior in high-yielding cows. No clear feeding behavioral effects of daily milking frequency were seen during the experimental period. Cabergoline induced decreased feeding behavior lasting approximately 24 h following administration, indicating collateral effects other than reduced prolactin secretion.
